# Uterine allograft removal by total laparoscopic hysterectomy after successful cesarean delivery in a living-donor uterus recipient with uterovaginal agenesis (MRKHS)

**DOI:** 10.1007/s00404-022-06796-7

**Published:** 2022-11-07

**Authors:** Sara Yvonne Brucker, Bernhard Krämer, Harald Abele, Melanie Henes, Markus Hoopmann, Dorit Schöller, Alfred Königsrainer, Hans Bösmüller, Konstantin Nikolaou, Patrick Krumm, Peter Rosenberger, Eckhard Heim, Bastian Amend, Steffen Rausch, Karina Althaus, Tamam Bakchoul, Martina Guthoff, Nils Heyne, Silvio Nadalin, Kristin Katharina Rall

**Affiliations:** 1 Tübingen University Women’s Hospital, Calwerstr. 7, 72076 Tübingen, Germany; 2 Department of General, Visceral, and Transplant Surgery, Tübingen University Hospital, 72076 Tübingen, Germany; 3 Institute of Pathology and Neuropathology, Tübingen University Hospital, 72076 Tübingen, Germany; 4 Department of Diagnostic and Interventional Radiology, University of Tübingen, 72076 Tübingen, Germany; 5 Department of Anesthesiology and Intensive Care, Tübingen University Hospital, 72076 Tübingen, Germany; 6 Department of Urology, Tübingen University Hospital, 72076 Tübingen, Germany; 7 Center for Transfusion Medicine, Tübingen University Hospital, 72076 Tübingen, Germany; 8 Section of Nephrology and Hypertension, Department of Diabetology, Endocrinology, and Nephrology, Tübingen University Hospital, 72076 Tübingen, Germany

**Keywords:** Uterus transplantectomy, Uterine allograft hysterectomy, Total laparoscopic hysterectomy, Living-donor uterus transplantation, Minimally invasive gynecological surgery, Congenital malformation of female genital tract

## Abstract

**Purpose:**

To limit the burden of long-term immunosuppression (IS) after uterus transplantation (UTx), removal of the uterine allograft is indicated after maximum two pregnancies. Hitherto this has required graft hysterectomy by laparotomy. Our objective was to demonstrate, as a proof of concept, the feasibility of less traumatic transplantectomy by total laparoscopic hysterectomy (TLH).

**Patient:**

A 37-year-old woman with uterovaginal agenesis due to Mayer–Rokitansky–Küster–Hauser syndrome (MRKHS) who had undergone neovaginoplasty at age 19 years prior to living-donor (LD) UTx in 10/2019 at age 35 years gave birth to a healthy boy by primary cesarean section in 06/2021. During pregnancy, she developed impaired renal function, with bilateral hydronephrosis, necessitating early allograft removal in 09/2021 to prevent chronic kidney disease, particularly during a potential second pregnancy.

**Methods:**

Transplantectomy by TLH essentially followed standard TLH procedures. We paid meticulous attention to removing as much donor tissue as possible to prevent postoperative complications from residual donor tissue after stopping IS, as well as long-term vascular damage.

**Results:**

TLH was performed successfully without the need to convert to open surgery. Surgical time was 90 min with minimal blood loss. No major complications occurred intra- or postoperatively and during the subsequent 9-month follow-up period. Kidney function normalized.

**Conclusions:**

To our knowledge, we report the first successful TLH-based removal of a uterine allograft in a primipara after LD UTx, thus demonstrating the feasibility of TLH in uterus recipients with MRKHS.

## What does this study add to the clinical work

**Table:** 

To limit the burden of long-term immunosuppression after uterus transplantation, removal of the uterine allograft is indicated after ≤2 pregnancies. While hitherto graft hysterectomy has been performed by laparotomy, our proof-of-concept study is the first to demonstrate that transplantectomy can be accomplished less traumatically by total laparoscopic hysterectomy.	

## Introduction

Since the first livebirth after human living-donor uterus transplantation (LD UTx) was performed in 2014 [1], a route to pregnancy and biological motherhood has opened up to women with absolute uterine factor infertility (AUFI) due to the absence of a functional uterus [2, 3]. This occurs particularly in women with congenital uterovaginal agenesis due to Mayer–Rokitansky–Küster–Hauser syndrome (MRKHs) [4].

UTx necessitates long-term immunosuppression (IS), as is the case in allogeneic solid organ transplantation in general. However, as a nonvital organ, the uterus is dispensable and hence the allograft should be removed again after a maximum of 2 births to limit the health-damaging effects of long-term IS. Hitherto uterine allograft removal usually has been done by open laparotomy, either immediately after cesarean delivery or in a later procedure [5]. Laparotomy has been considered to best ensure the complete removal of donor tissue—an absolute prerequisite for terminating IS treatment—because from an open surgery perspective it seemed easier and the risk of organ injury seemed to be lower by removing all donor tissue via laparotomy, especially in the area of the vascular anastomoses, the ureters, and the bladder. But since it was first reported in 1989 [6], total laparoscopic hysterectomy (TLH), a so-called minimally invasive procedure, has proved comparable or superior to abdominal hysterectomy in terms of intra- and postoperative complications and clearly superior in terms of reducing morbidity, duration of hospital stay, and convalescence time in both benign and malignant uterine disease [7, 8]. Especially in oncologic surgery, surgically complex lymph node dissection by laparoscopy in the area of the pelvic vessels and the ureters has been demonstrated to be technically feasible and as safe as open surgery in experienced hands in terms of intraoperative complications and organ and vessel injury. With these advantages in mind and backed by years of experience with TLH and the option of converting to laparotomy if required, our team decided to attempt to apply this technique to allograft removal after successful UTx and pregnancy.

Thus, our objective was to provide proof of concept that uterine allograft removal can be accomplished using the less traumatic TLH technique.

## Patients and methods

### Patient

Our patient, a premenopausal woman aged 37 years at the time of uterine allograft removal, was diagnosed as a teenager with uterovaginal agenesis due to type 1 MRKHS (= type A MRKHS, i.e., without any additional malformation) in the presence of a normal female 46,XX karyotype. At age 19 years, she underwent laparoscopically assisted neovaginoplasty as described by Brucker et al. [9, 10] at a specialist center abroad. Table 1 summarizes the patient’s baseline demographic and clinical characteristics prior to graft removal.

**Table 1:** Patient characteristics at baseline, uterus transplantation (UTx), pregnancy, and childbirth

Pre-UTx baseline	
Indication for UTx	Type 1 MRKHS
Menopausal status	Premenopausal
BMI, kg/m^2^	19.0
Smoking status	Nonsmoker
Age at neovagina creation, years	19
Neovaginal length, cm	9–10
Blood group	A Rh +
CMV status (recipient/donor)	pos/pos
HLA antibody screen	Negative, no DSA
HLA mismatches	4/6 for HLA class I; 2/4 for class II
HLA mismatches with potential father	2 repeated mismatches for HLA classes I and II
MRA: left/right uterine artery diameter, mm	3–4/2–5
MRA: left/right uterine vein diameter, mm	3/5
Recipient oocytes fertilized and cryopreserved for IVF preoperatively	14
**UTx surgery**	
Age at UTx, years	35
Recipient’s relationship to donor	Sister
UTx, month/year	10/2019
Surgical time for recipient, hours	9.19
Blood vessels used for anastomosis	*Left side*: –DUA (D) E/S onto EIA (R) –Uterine branch of UOV (D) with anastomosis onto DUV (D) onto EIV (R) *Right side*: –DUA (D) onto EIA (R) –DUV (D) and uterine branch of UOV (D) both E/S onto EIV (R) (UOV cranially from DUV)
Total ischemia time^a^, min	175
Warm ischemia time^b^, min	83
Estimated blood loss, mL	500
Surgical complications	Intraoperative reanastomosis of right DUV
Hospital stay, days	15
**Pregnancy and childbirth**	
First menstruation after UTx, weeks	5
Graft rejection, treatment	Mild rejection at 8 and 11 months post-UTx, treated with an additional 500 mg/day prednisolone for 3 days
Other postoperative events	Bilateral obstructive uropathy during pregnancy; CMV reactivation in pregnancy week 27 treated with valaciclovir
Embryo transfer	10/2020
Pregnancies after UTx	1
Deliveries after UTx	1
Date of delivery, month/year	06/2021
Mode of delivery	Primary cesarean section
Time from incision to delivery, min	9
Overall surgery time for delivery, min	79
Age at delivery, years	36
Gestational week + days at delivery	33 + 4
Placental histology	No definite sign of rejection or CMV infection, but omphalovasculitis, deciduitis, and chorioamnitis, hypotrophy according to gestational age

*MRKHS* Mayer–Rokitansky–Küster–Hauser syndrome; *BMI* Body mass index; *DSA* Preformed donor-specific anti-HLA antibody; *HLA* Human leukocyte antigen; MRA, Magnetic resonance angiography; *IVF* In vitro fertilization; *UTx* Uterus transplantation; DUA Deep uterine artery with internal iliac artery (IIA) Segment; E/S, end-to-side anastomosis; *D* donor; *EIA* External iliac artery; *R* recipient; *DUV* Deep uterine vein with internal iliac vein (IIV) segment; *UOV* Utero-ovarian vein; *EIV* External iliac vein; *CMV* Cytomegalovirus

^a^Total ischemia time = cold ischemia time, i.e., time from donor organ clamping to reperfusion

^b^Warm ischemia time = time from graft placement in the recipient until reperfusion; warm ischemia time is part of total ischemia time

In 2019, our patient and her uterus donor, her 32-year-old younger sister who had had two children, at age 25 and 27 years [11], completed their comprehensive eligibility evaluation according to our rigorous screening protocol for inclusion in the Tübingen UTx program as recently reported [12, 13]. UTx took place successfully in October 2019, followed by IS treatment, acetyl salicylic acid until delivery and dalteparin for the first 3 months, and a 6-month course of oral cotrimoxazole for *Pneumocystis jirovecii* prophylaxis. As an antiviral (cytomegalovirus, CMV) prophylaxis, valganciclovir 450 mg once daily was given for the first 3 months after UTx, as the uterus recipient tested CMV positive.

Post-UTx IS was instituted based on triple-drug combination regimens used in kidney transplant recipients, as previously reported in detail [11]. In brief, a 3-day induction therapy with antithymocyte globulin was initiated in parallel with a triple-drug regimen of tacrolimus (TAC), mycophenolate mofetil (MMF), and prednisolone. After 3 months, due to persistent and disabling tremor, TAC was replaced with the less neurotoxic drug ciclosporin in combination with MMF and prednisolone for 4 months before switching to the maintenance regimen consisting of ciclosporin, prednisolone, and azathioprine (AZA) as a replacement for the potentially teratogenic MMF.

Cervical biopsies to exclude graft rejection were performed 2x/week during the first month, 1x/week during months 2 and 3, every 2 weeks during months 4–6, and 1x/month during months 7–11 after UTx. The biopsies at 8 and 11 months revealed very mild rejection, which resolved after treatment with an additional 500 mg/day prednisolone for 3 days.

In vitro fertilization (IVF) of patient oocytes performed at another center in 2015 by intracytoplasmic sperm injection had yielded 14 cryopreserved pronuclear embryos [11]. The first and only embryo transfer (ET) was performed in 10/2020, 12 months after UTx. Pregnancy resulted in preterm delivery of a healthy boy by primary cesarean section at gestational week 33 + 4 days in 06/2021 due to vaginal bleeding and increased ductus venosus resistance in conjunction with known fetal intrauterine growth restriction. The newborn had a birthweight of 1635 g (8th percentile), a crown–heel length of 43 cm (20th percentile), a head circumference of 30 cm (15th percentile), and an APGAR score of 8/9/9, and he was CMV negative. Respiratory maladaptation necessitated treatment with continuous positive airway pressure for 13 h. Body weight at planned hospital discharge (week 36 + 0 days) was 1998 g (< 3rd percentile), crown–heel length was 45 cm (6th), and head circumferences was 31 cm (4th). The respective values (and percentiles) at age 6 months were 7740 g (40th), 65.5 cm (< 10th), and 42 cm (40th), and 9550 g (50th), 75 cm (25th), and 45,5 cm (45th) at age 12 months.

**Fig. 1:**
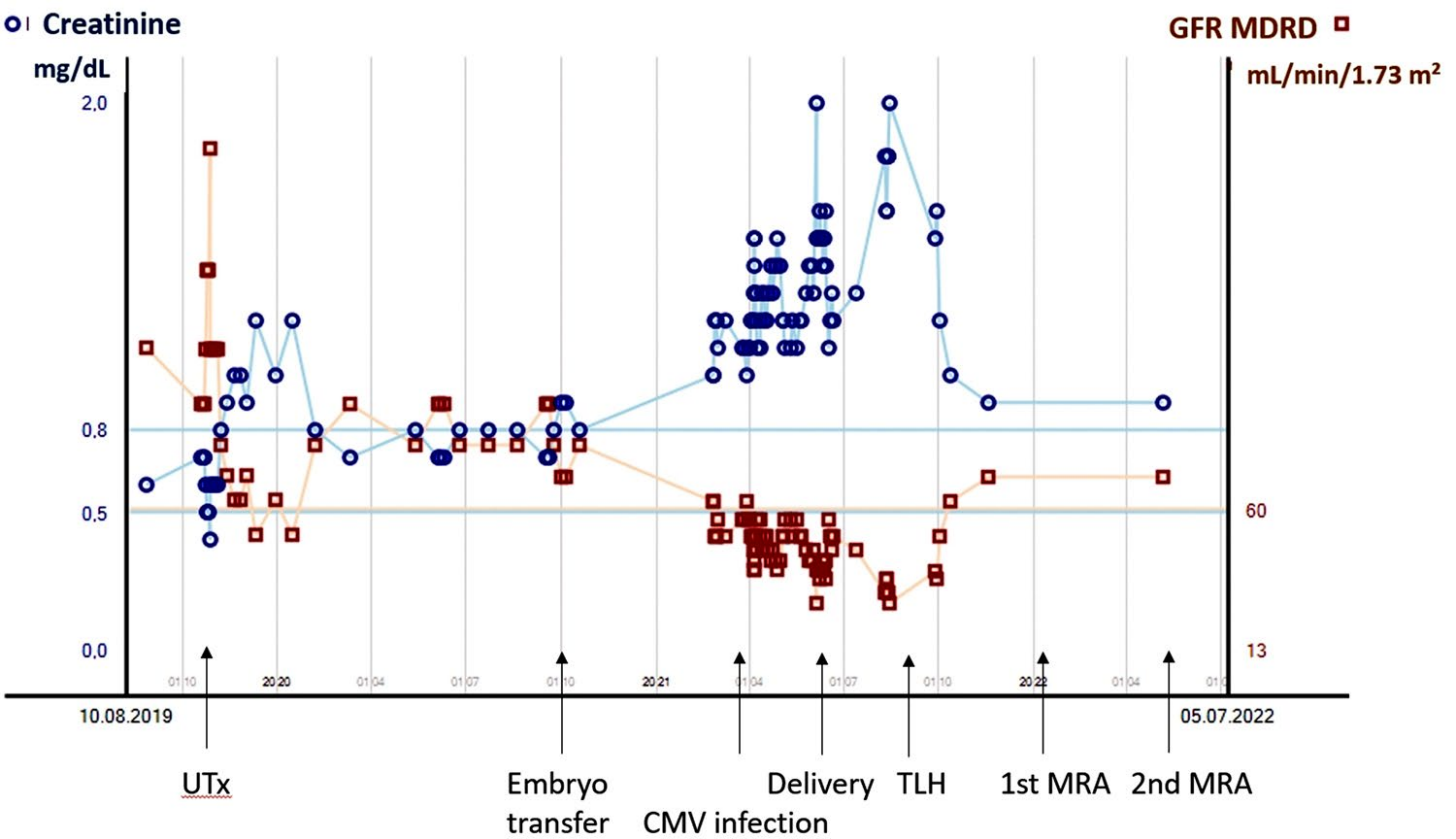
Time courses of the patient’s creatinine levels and glomerular filtration rates according to the Modification of Diet in Renal Disease (MDRD) formula (GFR MDRD) during the period from before UTx through June 2022

Before pregnancy, the patient’s post-UTx phase was unremarkable; however, at the end of the first trimester she then developed a severe complication, an impaired renal function probably based on preexisting bilateral ampullary renal pelvis. This started early into pregnancy, at gestational week 20/21, when our patient’s renal function declined as evidenced by increased renal retention levels with creatinine rising from baseline levels at ET of about 0.8 mg/dL to 1.2 mg/dL and a respective drop in glomerular filtration rate (GFR, with Modification of Diet in Renal Disease (MDRD)) from baseline levels at ET of about 80 mL/min/1.73 m^2^ to 51 mL/min/1.73 m^2^. Figure 1 shows the patient’s creatinine and GFR MDRD levels over time from before UTx through to allograft removal by TLH and beyond. Ultrasonography of the kidney revealed grade II bilateral hydronephrosis as evidenced by moderate distension of the renal pelvis and mild dilation but no thickening of the renal calyces. The cause may have been a known bilateral ureteropelvic junction obstruction, which had been diagnosed in the context of pyelonephritis 1 year before UTx. Treatment at the time consisted in the temporary bilateral insertion of double-J catheters. However, on removal of the double-J stents, the patient made a complete recovery and no further functional uropathy or renal function impairment occurred. At the time of evaluation for uterus transplantation, no relevant uropathy was visible on MRI apart from ampullary renal pelvis on both sides. However, the pregnancy-related increase in uterine volume caused obstructive uropathy to develop bilaterally, which increased up to grade III in the course of the pregnancy. Urinalysis remained unremarkable at all times but kidney function declined progressively. We decided against any form of invasive urinary diversion, e.g., by means of double-J catheter insertion or even the creation of a nephrostomy tube, considering the risk of complications from such treatment during pregnancy and in view of the multifactorial potential etiology of renal function deterioration. Rejection as a further cause of complications was excluded by a cervical biopsy taken in the 20th week of gestation.

At the time of the anomaly scan, i.e., at the beginning of the 2nd trimester, hyperechogenic bowel and placentomegaly were noted. We suspected a secondary fetal CMV infection due to a maternal CMV reactivation. The virus was detected in the maternal urine, stool, blood, and vaginal discharge. Amniocentesis revealed however that, surprisingly, the fetus was not infected. Due to the disseminated maternal infection, antiviral therapy with valaciclovir 5 g/day was initiated. The medication was discontinued when the virus was no longer detectable in the maternal compartments. In addition, the maternal retention levels were altered. After cessation of the CMV treatment, renal function gradually improved and remained stable during pregnancy at an estimated GFR of about 50 mL/min/1.73 m^2^. Only at the time of delivery did creatinine levels rise to 2.0 mg/dL and GFR drop to 28 mL/min/1.73 m^2^ (see Fig. 1).

After giving birth to her first child, our patient was deemed at high risk for permanent chronic renal insufficiency due to the uropathy she developed during pregnancy and the additional nephrotoxic effects of IS. However, the patient wished to have a second child. Therefore, allograft hysterectomy was not performed in conjunction with cesarean delivery. Instead, we took the opportunity to check whether renal function could be improved significantly after pregnancy (when uterine volume decreases again) and by urinary diversion. Renal scintigraphy showed possible obstruction on the left side and only functional outflow delay on the right. There was also evidence of an intraparenchymal transport disorder. While retrograde ureterography revealed no relevant stenosis on the right side, the left ureter showed signs of mild ureteropelvic junction obstruction. We therefore placed a double-J stent on the left side two months after delivery to relieve potential ureteral obstruction. However, renal impairment did not improve sufficiently. Although renal congestion was no longer present, creatinine persisted at 1.8 mg/dL with a GFR of 36 mL/min/1.73 m^2^. Reactivation of CMV infection as the cause of the reduced renal function was ruled out by repeated virologic testing for CMV and BK virus, a polyomavirus known to cause nephropathy, chronic kidney failure, and, ultimately, transplant loss.

Allograft rejection was excluded by cervical biopsy. To assess the chronification of renal injury, a renal biopsy was performed 10 weeks after the cesarean section and 10 days after placement of the double-J catheter on the left side with decongested kidneys on both sides. Histology revealed moderate signs of acute tubular epithelial damage, the presence of Tamm–Horsfall protein in tubuli, and discrete chronic interstitial inflammatory reaction. The degree of tubular atrophy and fibrosis was 25–30%. These changes were attributed to obstructive uropathy during pregnancy. Furthermore, mild arterial hyalinosis was detectable as a result of calcineurin inhibitor (CNI) toxicity. Hence, a second pregnancy was considered not to be medically responsible as the risk was deemed too high that the patient would again develop kidney failure, potentially necessitating kidney transplantation. After multiple consultations with the patient, our multidisciplinary transplantation board decided that the risk of chronic kidney disease, particularly if obstructive uropathy was to recur during a second pregnancy, constituted an absolute indication for removal of the uterine allograft.

### Transplantectomy

Minimally invasive removal of the uterine allograft was performed in 09/2021, 16 weeks after delivery by primary cesarean section in 06/2021, essentially following the detailed standard protocol for laparoscopic hysterectomies established at our institution [14]. The procedure was performed as follows.

#### Preoperative steps

One day before TLH, a double-J stent was prophylactically inserted into the right ureter. As mentioned above, the double-J stent in the left ureter was already in place. Preoperatively, the patient received cephalosporin for antibiotic prophylaxis < 30 min before surgery and low molecular weight heparin for thrombosis prevention. For surgery, the patient was placed in the lithotomy (Trendelenburg) position, prepped, and draped in the usual fashion, and placed under general anesthesia with endotracheal intubation.

Physical and vaginal examination findings were normal and the vagino-vaginal anastomosis was visible and without stenosis, the cervix was well formed, and the cervical canal was visible. On rectovaginal palpation, the uterus was mobile and normal in size without any palpable pathological resistance in the adnexal areas or evidence of uterine prolapse.

To enable transumbilical laparoscopy, a vertical transumbilical incision was made with an 11-mm scalpel, a Veress needle was inserted, and carbon dioxide was insufflated at an initial pressure of 20 mmHg to create a pneumoperitoneum. The pressure was subsequently lowered to 12 mmHg. Upon removal of the Veress needle and insertion of a 10 mm optical trocar, the upper and middle abdomen were inspected laparoscopically and revealed no remarkable findings, the vermiform appendix having a normal appearance.

A second trocar incision was made midline under diaphanoscopy without any complication at the site of the two previous midline laparotomies (UTx and cesarean delivery). A third and a fourth incision on either side were made lateral to the epigastric vessels, again under diaphanoscopy. The uterine manipulator (Hohl manipulator) with a portio adapter was inserted under laparoscopic view.

On visualization of the pelvic anatomical structures, the uterus exhibited only minimal filmy adhesions in the area of the anterior uterine wall and an intestinal adhesion on the left side in the mid-abdominal region at the level of the ovary. The latter was normally gyrated with signs of activity. There was a visible fimbrial end but no fallopian tube or connection to the patient’s round ligament of the uterus.

The anastomosis sutures for uterus fixation were discernable in the area of the two uterosacral ligaments and round ligaments. Uterine rudiments approx. 3–4 cm in size were present on both sides. The right uterine rudiment and the corresponding groin were also still fixed with the suture to the round ligament of the uterine allograft. The right ovary was in the normal anatomical position, clearly situated in a more caudal direction than the left ovary with a normal fimbrial end and the fallopian tube. The site of the uterotomy performed during delivery by cesarean section was visible and no niche was seen.

The bladder was retrogradely filled with 200 mL saline solution via the transurethral catheter. A good view of the transition between the recipient’s and the donor’s bladder peritoneum in the area of the Hohl adapter cap rendered a more extensive preparation of the recipient’s bladder unnecessary. Lysis of an adhesion between the colon/sigmoid colon and the pelvic wall was performed by sharp dissection. No bleeding occurred.

Both ureters were easily identified on account of the double-J stents. Both ureters were not dilated and clearly distant from the vascular anastomoses. The uterine artery anastomoses onto the respective external iliac artery were visible on both sides. The uterine artery itself was relatively prominent on either side. Both uterine veins used for anastomosis to the respective external iliac vein were readily visualizable.

#### Total laparoscopic hysterectomy

The hysterectomy as such began with the exposure of the pelvic walls and round ligaments of the uterus in the area of the suture fixation. The right and left round ligaments were coagulated and dissected on both sides. Preparation of the broad ligament of the uterus on both sides followed. The uterine artery and vein were visualized on both sides, sealed with two clips on the side of the external iliac vessels, coagulated on the side of the uterus, and then sharply divided, bilaterally resulting in only minimal vessel stumps of the uterine arteries, which were left in place. The vesicouterine fold was then cut and the bladder pushed minimally in the caudal direction along the Hohl manipulator. The monopolar needle was inserted via the middle trocar and the uterus subsequently detached from the neovagina. The neovagina was opened above the cap of the Hohl manipulator.

During the subsequent vaginal phase, the uterine allograft was extracted as atraumatically as possible. This was accomplished by grasping the uterus and carefully retrieving it from the abdominal cavity via the neovagina. The neovaginal vault was then closed by means of Vicryl-0 single-knot sutures via the vaginal route. Closure was complete and there were no signs of dehiscence. Rectal examination was unremarkable.

Both ureters were visible laparoscopically. There was no evidence of bleeding or injury to any adjacent organ. Careful intraabdominal wound toilet was performed and artificial ascites (physiological saline solution) was instilled for adhesion prophylaxis. Rectovaginal examination was unremarkable. A 100 mg diclofenac suppository was administered.

During the critical stages of transplantectomy, we paid meticulous attention to the removal of donor tissue to the largest surgically possible extent (up to about 5–10 mm of the graft vessels remaining in place) and restoration of the patient’s blood vessels to prevent the potential postoperative rejection of residual donor tissue, aneurism formation, and major bleeding. As there was no injury to the bladder or ureters on either side, we extracted both double-J catheters cystoscopically immediately after surgery.

### Postoperative care

Perioperatively, clindamycin was used as antibiotic prophylaxis. Further postoperative care included renal sonography, assessment of renal function, and renal scintigraphy. IS treatment with TAC and AZA was discontinued postoperatively on the day of allograft removal. Steroids were tapered down within 4 weeks after surgery.

Human leukocyte antigen (HLA) antibodies were determined two days before removal of the uterine allograft and monitored at discharge, 14 days to 4 weeks after transplantectomy, and 3 months postoperatively, with further follow-up determinations planned for 6 and 12 months after surgery. Magnetic resonance angiography was also performed 4 and 7 months after TLH-based transplantectomy to exclude any aneurism formation at the external iliac arteries.

#### Specimen analysis

The explanted uterine allograft and uterine tissue samples were sent to our pathology and virology departments for histologic analysis and CMV analysis, respectively. Serial monitoring of HLA class I and class II antibodies were measured by Luminex® screening (LABScreen™, One Lambda Inc., Canoga Park, CA, U.S.A.). Positive results were followed up with a single-antigen screening. All sera were treated with EDTA to exclude interference and the prozone effect. All mean fluorescence intensity levels > 1000 were included and recorded.

## Results

**Fig. 2:**
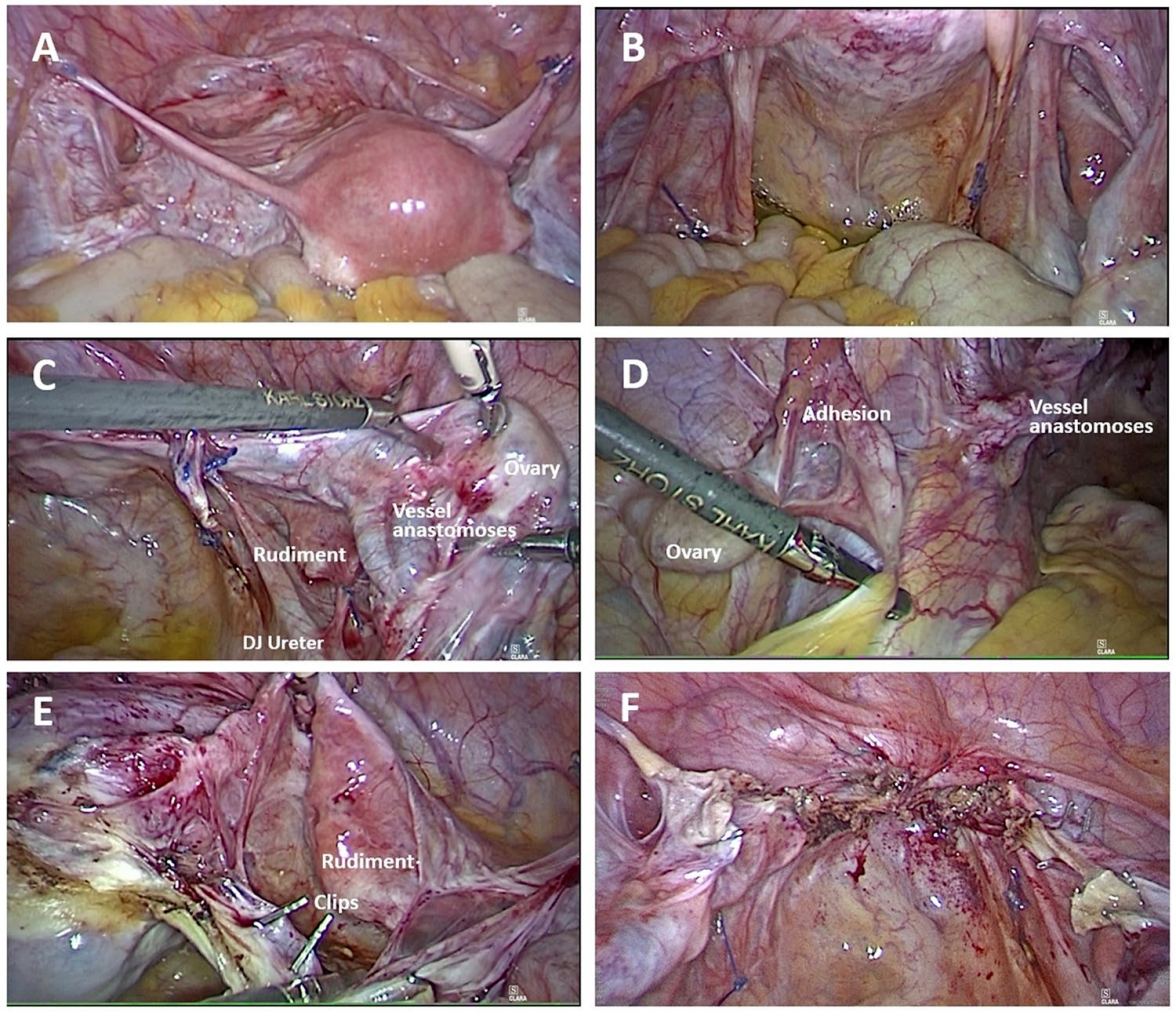
Intraoperative images. **A** View of the anterior uterine wall with fixation sutures in the region of the round ligaments on both sides; **B** view of the posterior uterine wall, pouch of Douglas with fixation sutures in the region of the uterosacral ligaments on both sides; **C** vascular anastomoses in the region of the iliac vessels on the right side, uterine rudiment and ovary on the right side, and the double-J stent in the right ureter; **D** vascular anastomoses in the region of the iliac artery on the left side, ovary on the left side, and discrete adhesions; **E** clips in the area of the dissection site of the uterine vessels on the right side; and **F** view after removal of the graft and closure of the vagina

TLH was performed successfully without the need to convert to open surgery. Figure 2 shows the laparoscopic surgical site prior to and after removal of the uterine graft. Surgical time was 90 min with minimal blood loss. No major complications occurred intra- or postoperatively or during the subsequent 9-month follow-up period, as of 30 June 2022. As shown in Fig. 1, the patient’s creatinine and GFR MDRD levels improved after TLH, remaining stable through June 2022.

**Fig. 3:**
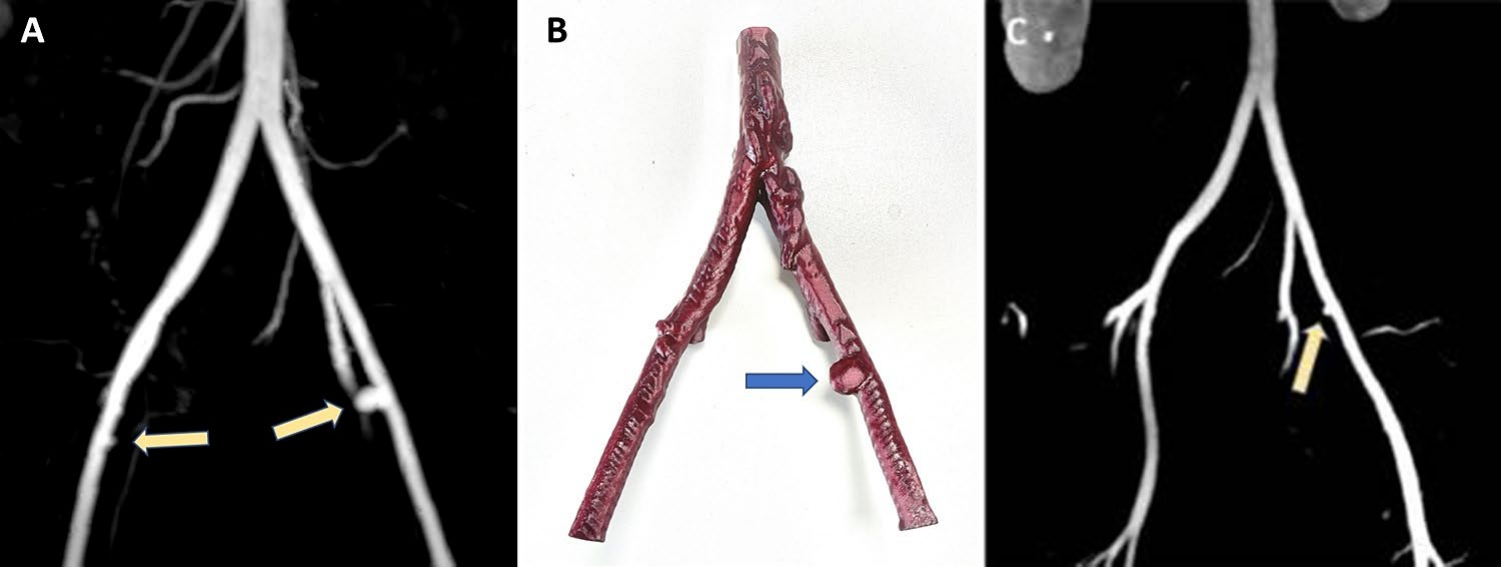
Magnetic resonance angiography after TLH-based uterine allograft removal. **A** MRA scan performed at the 4-month follow-up indicating postoperative arterial stumps on the external iliac arteries, 11 and 3 mm in diameter (arrows); **B** 3D printer-generated model of the patient’s aorta and common, external, and internal iliac arteries, generated from the scan shown in panel **A**; a vessel stump of 11 mm in diameter (arrow) at the anastomosis site on the external iliac artery (image of the 3D printed model generated by Jiri Fronek, M.D., Prague, Czech Republic); **C** MRA scan performed at the 7-month follow-up. Only a residual arterial stump (3 mm, arrow) on the left external iliac artery remained

Table 2 summarizes the key surgical data and results of postoperative analyses. Histologic examination of the explanted uterine graft, which weighed 100 g, revealed mild and focally moderate immune-modulated vasculopathy with endothelialiitis and obliterative changes, suggestive of chronic vascular rejection. Preexisting sclerosis of the major uterine arteries was also observed. As shown in Fig. 3, MRA scans indicated postoperative arterial stumps on both external iliac arteries of 11 (left side) and 3 mm (right side) in diameter at 4 months’ follow-up, which exhibited regression at 7 months’ follow-up, showing only a residual arterial stump of 3 mm on the left external iliac artery.

**Table 2:** Surgical and postoperative details of uterine allograft removal by total laparoscopic hysterectomy

Uterine allograft removal	
Indication for transplantectomy	Persistent renal impairment due to bilateral obstructive uropathy during pregnancy under immunosuppression
Removal of the uterine allograft, month/year	09/2021
Hysterectomy method	Total laparoscopic hysterectomy
Weight of explant, g	100
Surgery time, min	90
Estimated blood loss, mL	< 20 (insignificant)
Postoperative assessments and characteristics	
CMV analysis	PCR negative
Histology	Endothelialiitis, immune-modulated obliterative vasculopathy, chronic vascular rejection
Hospital stay, days	5

*CMV* Cytomegalovirus; *PCR* Polymerase chain reaction

Screening of HLA class I and class II antibodies was negative until months 3 after allograft TLH, when the recipient developed de novo donor-specific antibodies (DSA) against both the uterus donor and the father’s child. Among the detected HLA class I antibodies anti-A2, anti-A24 and anti-B51 were directed against the explanted uterus and anti-A1, anti-A24 and anti-B44 were directed against the child’s father. In class II, anti-DR4 and anti-D53 were directed against the explanted uterus.

## Discussion

We here report, as a proof of concept, the first minimally invasive removal worldwide of a uterine allograft using a TLH technique adapted to the specific requirements of allograft removal and subsequent termination of immunosuppressive therapy. Conversion to laparotomy was not required.

LD UTx in combination with IVF was first successfully performed in Sweden in 2013, resulting in the first healthy child worldwide being born to an LD UTx patient in 2014 [1, 15]. The first LD UTx in Germany was performed in 2016, followed by another three surgically successful transplantations, one in 2017 and two in 2019 [11], and one attempt which was aborted due to poor donor organ quality before initiating implantation surgery [12]. All four uterus recipients had their first menstruation within weeks of UTx and, after receiving IVF treatment and undergoing embryo transfer as previously described [11], gave birth to a healthy child by cesarean section. As of June 2022, a healthy child has been born to each of the four uterine allograft recipients successfully treated at our hospital.

While the advent of human LD UTx has opened up a route to biological motherhood for women with AUFI [3, 16], particularly those with MRKHS, it is currently considered necessary to remove the uterine allograft, generally after a second pregnancy at the latest. This is possible since UTx is not a life-saving procedure and done to limit the duration of IS therapy and its detrimental side effects on the body [17–19], such as increased risk of infection, renal dysfunction due to nephrotoxicity, malignancy, and the costs of long-term immunosuppressive drug treatment [18]. Thus, initial general recommendation was that hysterectomy should be performed after a recipient graft time (RGT) of 7 years or less, after live birth(s) as the goal of successful UTx have occurred [5]. However, it may become necessary in individual cases to remove the uterine allograft even before a pregnancy occurs in order to prevent potential harm to the recipient, as extensively discussed by Ayoubi and colleagues [20].

Thus, determining the timing for uterine graft removal on an individual basis is a complex process involving not only medical considerations such as immunosuppression, rejection, maternal and obstetrical complications, and RGT, but also the preferences and priorities the uterus recipient and her partner may have [17, 18]. While RGT in UTx patients is currently limited generally to about 5–7 years in most UTx trials [17, 21], it can be considerably longer as in the case of a surgically successful deceased-donor UTx reported from Turkey in 2011, in which the recipient experienced multiple embryo transfer failures and miscarriages, before delivering a healthy child 9 years after UTx [22].

Currently, uterine graft hysterectomy is performed at the same time as the cesarean section for the second child. However, graft removal may become necessary as a “stand-alone” procedure for medical reasons even before a successful pregnancy occurs. Such reasons include graft failure, repeated implantation failure, miscarriage, or other medical issues potentially impacting the recipient’s health, in particular kidney injury or severe infectious complications [5, 17]. Furthermore, graft hysterectomy may become necessary before a second pregnancy is pursued if medical problems or maternal complications during the first pregnancy pose a contraindication to further pregnancies, particularly in the event of renal function impairment caused by severe preeclampsia. This is more likely if the patient has only one kidney [21], currently a reason for exclusion from UTx, or ureteral stenosis, as was the case in our patient. Johannesson et al. also advised recipients who developed gestational diabetes during their first pregnancy against pursuing a second pregnancy [17]. Nonmedical reasons for uterine graft removal include the recipient’s or the couple’s choice or preference not to opt for a second child. In such cases the hysterectomy is not normally planned before 3–6 months after the first delivery so as to ensure the newborn is developing normally and is in good health before graft removal is performed. In any event, determining the timing for uterine graft removal and whether to perform the hysterectomy as a “stand-alone” procedure or in conjunction with the cesarean section remains the subject of debate for the following reasons.

While graft removal at the time of cesarean delivery in principle obviates the need for later “stand-alone” surgery, hysterectomy in conjunction with delivery bears the risk of severe complications. These include increased blood loss due to the enlarged size of the gravid and post-delivery uterus and the dilated uterine vessels, and potential iatrogenic damage to adjacent structures, e.g., the ureters and the bladder. Adhesions are less of a problem as immunosuppression mitigates their formation. The main surgical challenges of allograft hysterectomy relate not so much to the preceding UTx surgery as to the restoration of the iliac vessels. These considerations favor separate removal of the uterine allograft at a later time [17].

To the patient, delaying allograft hysterectomy until several months after cesarean delivery obviously means experiencing renewed surgical trauma requiring an additional recovery period while having to nurse her infant, as was the case in our patient. However, delaying the irreversible step of allograft removal also offers decisive advantages in that it enables monitoring the newborn’s viability and development over the first few months of life. For instance, sudden infant death soon after delivery would aggravate the emotional impact of child loss on the mother if the allograft had been removed at the time of cesarean delivery. These considerations beg the question as to the feasibility of “stand-alone” graft hysterectomy by TLH in patients in whom graft removal is not performed in conjunction with cesarean delivery, thus utilizing the known advantages of minimally invasive surgery over laparotomy, in particular reduced postoperative morbidity and shorter convalescence [8].

As of June 2022, three out of our four uterus recipients still have their uterine allograft in place after their first deliveries and are eligible for a second pregnancy. In the patient we report on here, however, the hysterectomy became necessary after the first birth due to the risk of long-term renal failure. Removal of the uterine allograft was not performed in conjunction with the patient’s first cesarean delivery as there was a chance she might make a full recovery from the uropathy, which would have enabled a second pregnancy.

Although the laboratory studies and clinical renal function values improved postpartum, and unilateral insertion of a double-J stent into the left ureter did resolve hydronephrosis, the renal biopsy showed incipient mild damage, indicating that renewed obstructive uropathy would likely result in aggravated renal damage in the longer term. It was considered likely that these problems might recur during a subsequent pregnancy, since the patient had a preexisting ureteral stenosis, which was masked at the time of UTx. The risk of severe renal impairment recurring might be further exacerbated by the mandatory IS drugs, as CNIs are potentially nephrotoxic. Dilation of the renal pyelocaliceal system (RPCS) is observed in the vast majority of all pregnant women. Depending on cohort, gestational age, and the number of previous pregnancies, it occurs in up to 90% of women and can therefore be considered physiological [23]. If pregnancy is the sole cause of the obstruction, RPCS dilation is considered pregnancy-associated. In this case, RPCS dilation does not result in hydronephrosis and is fully reversible as pregnancy-associated urinary retention by 6 months postpartum at the latest. The highest incidence of RPCS dilation appears to occur around week 28 of pregnancy. However, in the presence of additional risk factors, recurrence of pregnancy-associated hydronephrosis may evolve into chronic renal insufficiency.

Chronic kidney disease could potentially end in terminal renal failure with the consequent potential need for kidney transplantation. Moreover, since the patient had already received an organ transplant and could therefore have become sensitized with donor-specific HLA antibodies—which in fact she did after the hysterectomy—the pool of potential kidney donors for a second organ transplantation would have been massively reduced. Therefore, we focused on avoiding any risk of further deterioration of renal function that could have ultimately ended in a kidney transplantation.

For the above reasons, we considered the risks potentially associated with a second pregnancy to be too high to be medically responsible and therefore advocated removal of the uterine allograft after the patient’s first delivery but not before completion of the postpartum period. Graft removal thus needed to be performed as a “stand-alone” surgery after cesarean delivery of the first child. We then considered this also to be possible by laparoscopy, as the convalescence period was likely to be much shorter than after abdominal surgery.

Since the first laparoscopic hysterectomy for benign indications was reported in 1989 [6], TLH has become established worldwide for benign uterine disease and has proved comparable or superior to the open procedure in terms of intra- and postoperative complications, and clearly superior in terms of reducing morbidity, duration of hospital stay, and convalescence time [7, 8].

For malignant uterine cancer, which requires more extensive dissection, especially along the iliac vessels and the ureters, minimally invasive access in the form of total radical laparoscopic hysterectomy (TLRH) also has been found not to be associated with increased complication rates. For instance, a meta-analysis of peri- and postoperative morbidities and complications comparing robotic and laparoscopic radical hysterectomy with open surgery showed that minimally invasive radical hysterectomy appears to be associated with reduced intraoperative morbidity and blood loss and improved convalescence after surgery [24].

A retrospective cohort study analyzing a database for 2008–2014 with patients who had undergone surgery for endometrial cancer showed that adoption of minimally invasive surgery is associated with substantial decreases in 30-day morbidity, readmission, and reoperation for women treated for endometrial cancer in the United States [25].

Minimally invasive surgery can be used even in complex situations like in carcinosarcoma or interval debulking after neoadjuvant chemotherapy in patients with advanced ovarian cancer [26, 27]. Thus, when routinely performed, even operations involving extensive preparation and dissection in narrow spaces and around crucial structures such as vessels, ureters, and bowels, minimally invasive access is possible and may even be superior to open surgery [28, 29]. Thus, when the minimally invasive approach is routinely employed, as is the case at our university hospital [30–34], even complex surgeries have a similar outcome but with the advantages of minimally invasive access.

Furthermore, minimally invasive techniques have already been used in UTx [3]. While in earlier uterus transplantations, all donor explantation and recipient implantation procedures were performed by laparotomy, more recently minimally invasive techniques have been introduced in both cases with the objective of reducing the long duration of donor surgery and the long recovery periods associated with laparotomy. Moreover, in light of recent advances in minimally invasive donor uterus procurement and even robotic uterus implantation [35], it seemed to us to be the next logical step also to consider minimally invasive graft removal.

So far, however, removal of LD allografts has always been performed by laparotomy, irrespective of the timing, i.e., before any delivery or as a later “stand-alone” procedure after a postpartum convalescence period [5, 36]. This has been based on the opinion that removal of donor tissue to the largest possible extent and minimization of the risk of injury to internal organs, the ureters, and blood vessels are best achieved by an open procedure, not least to avoid long-term complications such as aneurysm formation.

However, the prospect of a faster recovery due to considerably less surgical trauma and the expertise our gynecological team had in performing hysterectomies even in cases of severe benign or malignant gynecological disease provided a rationale for minimally invasive removal of the donated uterus. With our patient’s consent, we therefore decided to attempt the first-ever transplantectomy by TLH with the option of conversion to laparotomy if needed. To our knowledge, we here report the first minimally invasive removal worldwide of a transplanted uterus using an adapted TLH technique. Adaptation of standard TLH technique primarily pertained to vessel preparation involving the external vessels, such as is necessary in a radical hysterectomy for cervical cancer in that the uterine vessels need to be transected at the external iliac vessels and the bladder must also be dissected from the anterior cervical wall to the vagina, and thus, the ureters must also be exposed down to the entrance to the bladder.

In particular, laparoscopic removal of the uterine graft could prove difficult due to, essentially, two major considerations: (1) the potential presence of extensive adhesions and (2) the UTx-related altered anatomy of uterine vessels in relation to the position of the ureters [5, 20].

Adhesions and tissue scars at the interface between donor and recipient tissue encountered at this challenging operative site make preparation more demanding and can potentially result in increased complication rates of bladder and ureter injury, in particular. In addition, the altered anatomical position of the uterine vessels relative to the ureters and external iliac vessels needs to be observed. At the same time, complete resection of donor tissue is crucial to preventing sensitization and rejection reactions upon withdrawal of IS.

As a preventive measure, we placed double-J stents preoperatively to enable easy identification of, and avoid surgical trauma to, the ureters. Thus, even in the presence of extensive adhesion formation, which was not the case in our patient, the placement of the double-J stents allowed the ureters to be well demarcated, even where they were hidden among adhesions. Ureteral double-J stenting is also described in the literature, with studies reporting the placement of double-J stents prior to laparoscopic Wertheim surgery for cervical cancer [37] or complex gynecological surgery for endometriosis [38] and, more recently, hysterectomy after UTx [5]. Although a recent study found that laparoscopic hysterectomy may be associated with a higher risk of ureteral injury than abdominal hysterectomy, studies by Chang et al. [39] and Han et al. [40] comparing the clinical efficacy of a temporary ureteral catheter in cervical cancer patients undergoing laparoscopic radical hysterectomy showed that a ureteral catheter that is placed preoperatively can help to identify the ureter but does not reduce the incidence of ureteral injury.

Dissection and removal of the anastomoses in the region of the iliac vessel pose the other significant challenge. Here, the problems involved relate to the presence of neovascular plexuses and the untypical anatomy due to the anastomoses in the region of the externa vessels instead of the natural branching of the uterine vessels from the internal iliac vessels [20]. During implantation of the donor uterus at UTx the uterine vessels are joined to the larger external iliac vessels, resulting in an unusual position relative to the ureters. This needs to be taken into account when removing the graft. Therefore, adaptation is necessary with regard to the fact that especially in the area of the iliac vessels, preparation must be close to the anastomosis. Hence TLH is more similar to TLRH in terms of required surgical experience and skills, and challenges (cf. description of the surgical technique above). The key difference to a classical hysterectomy is that the uterine artery and vein were visualized on both sides, sealed with 2 clips on the side of the external iliac vessels, coagulated on the side of the uterus, and then sharply divided. Bilaterally this resulted in only minimal vessel stumps of the uterine arteries, which were left in place.

Dissection needs to be performed as close to the anastomosis as possible to achieve best possible removal of the donor tissue and avoid the risk of (iatrogenic) aneurysm formation when too large a vessel stump is left in place. Aneurism formation at vascular anastomosis sites has been reported as a rare complication after renal allograft nephrectomy [41–43]. In our patient, TLH as such was not associated with any major intra- or postoperative complications related to the procedure. In particular, no aneurisms of the anastomosed vessels occurred postoperatively in a long-term follow-up of 7 months.

The first minimally invasive TLH-based uterine allograft removal in a 37-year-old primiparous LD UTx recipient with MRKHS reported here demonstrates that TLH can be successfully performed in uterus recipients with MRKHS. This provides proof of concept that laparoscopic hysterectomy for uterine allograft removal is technically feasible and can be performed without intraoperative complications such as vascular or ureteral injury, bladder injury, or long-term complications such as aneurysm formation, as evidenced by our 4- and 7-month follow-up data. Our patient experienced no significant blood loss, (supravaginal) hematoma, urinary tract infection, or dehiscence of the vaginal stump. Postoperative vaginal examination showed a good anatomical and functional length and width, enabling unimpaired sexual intercourse.

The surgical time we achieved with TLH after UTx was 90 min, which compares favorably with abdominal total hysterectomy, which typically takes 3–5 h [5]. Other factors to be considered when comparing minimally invasive surgery versus laparotomy include the extent and consequences of incision, postoperative pain, length of hospital stay, psychological health, and quality of life [44]. Again, TLH after UTx compared favorably in our hands.

However, we did note the development of non-DSA HLA antibodies and HLA antibodies against the child’s father and DSA directed against the transplanted uterus with a current panel reactivity > 90% after graft removal and withdrawal of IS therapy. The reasons for this are unclear, but conceivably an unspecified amount of vascular tissue from the donor may have remained in the recipient, enough to elicit an immunological response after TLH and IS withdrawal. The question is, whether slower tapering of immunosuppression would have reduced antibody formation or just delayed their appearance in the blood. Our patient also developed antibodies against her partner’s HLA type, indicating that sensitization had occurred at least during childbirth, as is often seen in pregnant women. The detection of HLA antibodies as late as six months after TLH would then be indicative of the binding of antibodies to the uterus and the later suppression of antibody formation by immunosuppression, the latter being a mechanism that cannot be stopped permanently without intensive immunosuppression.

With other organs such as the kidney, when an allograft becomes functionally impaired and a new organ is required or dialysis needs to be reinstated, the original graft will most often be left in place in order to bind any antibodies that may have formed. However, unlike other organs such as the kidney, lung, or heart, which require life-long IS, the uterus is not a vital organ. Therefore, it should be removed once the objectives of UTx, i.e., pregnancy and childbirth, have been achieved.

Whether and to what extent donor-specific antibodies are formed after removal of the uterine graft, as was the case in our patient, warrants further investigation and analysis. The formation of donor-specific antibodies leads to a dramatic reduction of the pool of potential organ donors, should the patient later need to undergo kidney or heart transplantation. In the case of our patient, the pool would be reduced to about 3.6% of kidneys available from Eurotransplant.

This is a crucial aspect to be considered in the decision-making process in advance of UTx and on uterine graft hysterectomy in view of the inherent risk that kidney transplantation may prove impossible in highly sensitized uterus recipients due to the absence of an immunological match in the event of future renal failure. Fortunately, our patient regained normal kidney function to the extent that kidney transplantation appears unlikely as long as no further kidney damage occurs. However, patients, their partners, and donors need to be informed about this potential risk prior to UTx, and their physicians need to make every effort to provide maximum safety and protection for the recipient. However, this is of no consequence to the surgical procedure in terms of laparoscopic technique versus laparotomy. Uterine allograft removal by TLH is feasible and open surgery is not necessary to prevent HLA antibody formation.

## Conclusion

Our proof of concept demonstrates that TLH-based hysterectomy provides a reliable method to remove an LD uterine allograft after pregnancy and childbirth. Conversion to laparotomy was not necessary. The general benefits of reduced morbidity and shorter hospital stay were also seen in our patient. The general suitability and practicality of TLH in uterine allograft removal after LD UTx warrants further investigation in larger patient cohorts. Further experience with TLH-based uterine allograft removal also needs to be gained to improve the learning curve. Moreover, standard TLH-based graft hysterectomy needs to be compared with endoscopic transplantectomy by robotic surgery. Lastly, data on methods of UTx-related uterine allograft removal should be collected worldwide and deposited in an international registry, e.g., the registry established and maintained by the International Society of Uterus Transplantation (ISUTx) [16].

## Data Availability

Not applicable. No datasets were generated or analyzed in the present study.

## Abbreviations

AUFI: Absolute uterine factor infertility
AZA: Azathioprine
BMI: Body mass index
CMV: Cytomegalovirus
CNI: Calcineurin inhibitor
D: Donor
DSA: Preformed donor-specific anti-HLA antibody
DUA: Deep uterine artery
DUV: Deep uterine vein
E/S: End to side (anastomosis)
EIA: External iliac artery
EIV: External iliac vein
GFR: Glomerular filtration rate
GFR MDRD: Glomerular filtration rate according to the Modification of Diet in Renal Disease formula
HLA: Human leukocyte antigen
IIA: Internal iliac artery
IIV: Internal iliac vein
IS: Immunosuppression / immunosuppressive
ISUTx: International Society of Uterus Transplantation
IVF: In vitro fertilization
LD: Living donor
MMF: Mycophenolate mofetil
MRA: Magnetic resonance angiography
MRKHS: Mayer–Rokitansky–Küster–Hauser syndrome
R: Recipient
RGT: Recipient graft time
TAC: Tacrolimus
TLH: Total laparoscopic hysterectomy
UOV: Utero-ovarian vein
UTx: Uterus transplantation

## References

[1] Brännström M, Johannesson L, Bokström H, Kvarnström N, Mölne J, Dahm-Kähler P, Enskog A, Milenkovic M, Ekberg J, Diaz-Garcia C, Gäbel M, Hanafy A, Hagberg H, Olausson M, Nilsson L (2015). Livebirth after uterus transplantation. Lancet.

[2] Brännström M, Enskog A, Kvarnström N, Ayoubi JM, Dahm-Kähler P (2019). Global results of human uterus transplantation and strategies for pre-transplantation screening of donors. Fertil Steril.

[3] Brännström M, Kvarnström N, Dahm-Kähler P (2020). Novel approaches in uterus transplantation. Curr Opin Organ Transplant.

[4] Morcel K, Camborieux L, Guerrier D (2007). Mayer-Rokitansky-Küster-Hauser (MRKH) syndrome. Orphanet J Rare Dis.

[5] Karlsson CC, Dahm-Kähler P, Kvarnström N, Mölne J, Broecker V, Brännström M (2022). Hysterectomy after uterus transplantation and detailed analyses of graft failures. Acta Obstet Gynecol Scand.

[6] Reich H, DeCaprio J, McGlynn F (1989). Laparoscopic Hysterectomy. J Gynecol Surg.

[7] Müller A, Thiel FC, Renner SP, Winkler M, Häberle L, Beckmann MW (2010). Hysterectomy-a comparison of approaches. Dtsch Arztebl Int.

[8] Aarts JW, Nieboer TE, Johnson N, Tavender E, Garry R, Mol BW, Kluivers KB (2015). Surgical approach to hysterectomy for benign gynaecological disease. Cochrane Database Syst Rev.

[9] Brucker SY, Gegusch M, Zubke W, Rall K, Gauwerky JF, Wallwiener D (2008). Neovagina creation in vaginal agenesis: development of a new laparoscopic Vecchietti-based procedure and optimized instruments in a prospective comparative interventional study in 101 patients. Fertil Steril.

[10] Brucker SY, Rall K, Wallwiener D, Nezhat C, Nezhat FR, Nezhat C (2013). Laparoscopically assisted neovaginoplasty. Nezhat’s video-assisted and robotic-assisted laparoscopy and hysteroscopy.

[11] Brucker SY, Strowitzki T, Taran FA, Rall K, Schöller D, Hoopmann M, Henes M, Guthoff M, Heyne N, Zipfel S, Schäffeler N, Bösmüller H, Fend F, Rosenberger P, Heim E, Wiesing U, Nikolaou K, Fleischer S, Bakchoul T, Poets CF, Goelz R, Wiechers C, Kagan KO, Krämer B, Reisenauer C, Oberlechner E, Hübner S, Abele H, Dahm-Kähler P, Kvarnström N, Brännström M, Nadalin S, Wallwiener D, Königsrainer A (2020). Living-donor uterus transplantation: pre-, intra-, and postoperative parameters relevant to surgical success, pregnancy, and obstetrics with live births. J Clin Med.

[12] Brucker SY, Brännström M, Taran FA, Nadalin S, Königsrainer A, Rall K, Schöller D, Henes M, Bösmüller H, Fend F, Nikolaou K, Notohamiprodjo M, Rosenberger P, Grasshoff C, Heim E, Krämer B, Reisenauer C, Hoopmann M, Kagan KO, Dahm-Kähler P, Kvarnström N, Wallwiener D (2018). Selecting living donors for uterus transplantation: lessons learned from two transplantations resulting in menstrual functionality and another attempt, aborted after organ retrieval. Arch Gynecol Obstet.

[13] Taran FA, Schöller D, Rall K, Nadalin S, Königsrainer A, Henes M, Bösmüller H, Fend F, Nikolaou K, Notohamiprodjo M, Grasshoff C, Heim E, Zipfel S, Schäffeler N, Bakchoul T, Heyne N, Guthoff M, Krämer B, Reisenauer C, Hoopmann M, Kagan KO, Brännström M, Wallwiener D, Brucker SY (2019). Screening and evaluation of potential recipients and donors for living donor uterus transplantation: results from a single-center observational study. Fertil Steril.

[14] Wallwiener D, Becker S, Beckmann MW, Brucker SY, Friese K, Isaacson KB, Jonat W, Wattiez A, Wallwiener D, Becker S (2013). Laparoscopic hysterectomy. Atlas of gynecologic surgery.

[15] Brännström M, Bokström H, Dahm-Kähler P, Diaz-Garcia C, Ekberg J, Enskog A, Hagberg H, Johannesson L, Kvarnström N, Mölne J, Olausson M, Olofsson JI, Rodriguez-Wallberg K (2016). One uterus bridging three generations: first live birth after mother-to-daughter uterus transplantation. Fertil Steril.

[16] Brännström M, Tullius SG, Brucker S, Dahm-Kähler P, Flyckt R, Kisu I, Andraus W, Wei L, Carmona F, Ayoubi JM, Scollo P, Weyers S, Fronek J (2022). Registry of the international society of uterus transplantation: first report. Transplantation.

[17] Johannesson L, Wall A, Warren AM, Gregg AR, Testa G (2021). Decisions on second pregnancy after uterus transplantation and timing for removal of the uterus-DUETS (Dallas UtErus transplant study). BJOG.

[18] Kisu I, Banno K, Aoki D (2021). Re: Decisions on second pregnancy after uterus transplantation and timing for removal of the uterus-DUETS (Dallas UtErus transplant study). BJOG.

[19] Boulay H, Mazaud-Guittot S, Supervielle J, Chemouny JM, Dardier V, Lacroix A, Dion L, Vigneau C (2021). Maternal, foetal and child consequences of immunosuppressive drugs during pregnancy in women with organ transplant: a review. Clin Kidney J.

[20] Ayoubi JM, Carbonnel M, Pirtea P, Kvarnström N, Brannström M, Dahm-Kähler P (2019). Laparotomy or minimal invasive surgery in uterus transplantation: a comparison. Fertil Steril.

[21] Brännström M, Dahm-Kähler P, Kvarnström N, Enskog A, Olofsson JI, Olausson M, Mölne J, Akouri R, Järvholm S, Nilsson L, Stigson L, Hagberg H, Bokström H (2022). Reproductive, obstetric, and long-term health outcome after uterus transplantation: results of the first clinical trial. Fertil Steril.

[22] Ozkan O, Ozkan O, Dogan NU, Bahceci M, Mendilcioglu I, Boynukalin K, Ongun H, Kantarci AM, Yaprak M, Cengiz M, Hadimioglu N, Kafadar YT, Celik K (2022). Birth of a Healthy Baby 9 years after a surgically successful deceased donor uterus transplant. Ann Surg.

[23] Faúndes A, Brícola-Filho M, Pinto e Silva JL,  (1998). Dilatation of the urinary tract during pregnancy: proposal of a curve of maximal caliceal diameter by gestational age. Am J Obstet Gynecol.

[24] Kampers J, Gerhardt E, Sibbertsen P, Flock T, Hertel H, Klapdor R, Jentschke M, Hillemanns P (2021). Perioperative morbidity of different operative approaches in early cervical carcinoma: a systematic review and meta-analysis comparing minimally invasive versus open radical hysterectomy. Arch Gynecol Obstet.

[25] Casarin J, Multinu F, Ubl DS, Dowdy SC, Cliby WA, Glaser GE, Butler KA, Ghezzi F, Habermann EB, Mariani A (2018). Adoption of minimally invasive surgery and decrease in surgical morbidity for endometrial cancer treatment in the United States. Obstet Gynecol.

[26] Brown J, Drury L, Crane EK, Anderson WE, Tait DL, Higgins RV, Naumann RW (2019). When less is more: minimally invasive surgery compared with laparotomy for interval debulking after neoadjuvant chemotherapy in women with advanced ovarian cancer. J Minim Invasive Gynecol.

[27] Pedra Nobre S, Mueller JJ, Gardner GJ, Long Roche K, Brown CL, Soslow RA, Alektiar KM, Sonoda Y, Broach VA, Jewell EL, Zivanovic O, Chi DS, Abu-Rustum NR, Leitao MM (2020). Comparison of minimally invasive versus open surgery in the treatment of endometrial carcinosarcoma. Int J Gynecol Cancer.

[28] Zhou ZN, Chen L, Melamed A, St Clair CM, Hou JY, Khoury-Collado F, Gockley A, Hershman DL, Wright JD (2022). Adoption of minimally invasive surgery after neoadjuvant chemotherapy in women with metastatic uterine cancer. Gynecol Oncol.

[29] Kim NR, Lee AJ, Yang EJ, So KA, Lee SJ, Kim TJ, Shim SH (2022). Minimally invasive surgery versus open surgery in high-risk histologic endometrial cancer patients: a meta-analysis. Gynecol Oncol.

[30] Sahbai S, Taran FA, Staebler A, Wallwiener D, la Fougere C, Brucker S, Dittmann H (2017). Sentinel lymph node mapping using SPECT/CT and gamma probe in endometrial cancer: an analysis of parameters affecting detection rate. Eur J Nucl Med Mol Imaging.

[31] Rothmund R, Hartkopf A, Joachim C, Walter CB, Wallwiener M, Kraemer B, Brucker SY, Staebler A, Taran FA (2014). Clinical characteristics, pathological reevaluation, surgical management and adjuvant therapy of patients with endometrial stromal tumors. Arch Gynecol Obstet.

[32] Sahbai S, Taran FA, Fiz F, Staebler A, Becker S, Solomayer E, Wallwiener D, la Fougere C, Brucker S, Dittmann H (2016). Pericervical injection of 99mTc-nanocolloid is superior to peritumoral injection for sentinel lymph node detection of endometrial cancer in SPECT/CT. Clin Nucl Med.

[33] Brucker SY, Taran FA, Wallwiener D (2014). Sentinel lymph node mapping in endometrial cancer: a concept ready for clinical routine?. Arch Gynecol Obstet.

[34] Wagner P, Kommoss FKF, Kommoss S, Hartkopf AD, Pasternak I, Oberlechner E, Greif K, Wallwiener M, Neis F, Abele H, Kramer B, Reisenauer C, Staebler A, Wallwiener D, Brucker SY, Taran FA (2019). Unexpected malignant uterine pathology: Incidence, characteristics and outcome in a large single-center series of hysterectomies for presumed benign uterine disease. Gynecol Oncol.

[35] Dahm-Kähler P, Kvarnström N, Brännström M (2021). Robotic live donor hysterectomy. Curr Opin Organ Transplant.

[36] Brännström M, Belfort MA, Ayoubi JM (2021). Uterus transplantation worldwide: clinical activities and outcomes. Curr Opin Organ Transplant.

[37] Liang C, Liu P, Cui Z, Liang Z, Bin X, Lang J, Chen C (2020). Effect of laparoscopic versus abdominal radical hysterectomy on major surgical complications in women with stage IA-IIB cervical cancer in China, 2004–2015. Gynecol Oncol.

[38] Melnyk A, Rindos NB, El Khoudary SR, Lee TTM (2020). Comparison of laparoscopic hysterectomy in patients with endometriosis with and without an obliterated cul-de-sac. J Minim Invasive Gynecol.

[39] Chang EJ, Mandelbaum RS, Nusbaum DJ, Violette CJ, Matsushima K, Klar M, Matsuzaki S, Machida H, Kanao H, Roman LD, Matsuo K (2020). Vesicoureteral injury during benign hysterectomy: minimally invasive laparoscopic surgery versus laparotomy. J Minim Invasive Gynecol.

[40] Han L, Cao R, Jiang JY, Xi Y, Li XC, Yu GH (2014). Preset ureter catheter in laparoscopic radical hysterectomy of cervical cancer. Genet Mol Res.

[41] Diller R, Holzen J, Senninger N, Kramer S (2006). Interventional stenting for ruptured iliac aneurysm following transplant nephrectomy. Transplant Proc.

[42] Eng MM, Power RE, Hickey DP, Little DM (2006). Vascular complications of allograft nephrectomy. Eur J Vasc Endovasc Surg.

[43] Borges L, Oliveira N, Dias E, Cassio I (2014). Iliac artery pseudoaneurysm: a rare complication following allograft nephrectomy. BMJ Case Rep.

[44] Kvarnström N, Järvholm S, Johannesson L, Dahm-Kähler P, Olausson M, Brännström M (2017). Live donors of the initial observational study of uterus transplantation-psychological and medical follow-up until 1 year after surgery in the 9 cases. Transplantation.

